# Activation of TRAF1 induced by USP7/SP1 exacerbates the severity of infantile pneumonia

**DOI:** 10.1186/s41065-025-00410-x

**Published:** 2025-03-22

**Authors:** Ying Liu, Yilun Ji, Yu Zhang, Zhengsi Li

**Affiliations:** 1 Department of Child Healthcare, Northwest Women’s and Children’s Hospital, Xi’an, 710061 China; 2 Department of Child Healthcare, Xi’an People’s Hospital (Xi’an Fourth Hospital), No. 21, Jiefang Road, Xi’an, 710004 China; 3 Department of Anesthesiology, The Second Affiliated Hospital of Harbin Medical University, Harbin, 150086 China; 4 Department of Pediatrics, Mianyang Central Hospital, Mianyang, 621000 China

**Keywords:** Infantile pneumonia, TRAF1, USP7, SP1

## Abstract

**Background:**

Infantile pneumonia (IP) is a leading cause of morbidity and mortality in children worldwide, with limited treatment options. Tumor necrosis factor receptor-associated factor 1 (TRAF1) has been implicated in the pathogenesis of various inflammatory diseases. Given the lack of effective therapies in IP, understanding the role of TRAF1 in regulating IP is crucial for developing new therapeutic strategies.

**Methods:**

This study utilized in vitro and in vivo models to investigate the role of TRAF1 in IP. WI-38 cells were stimulated with lipopolysaccharide (LPS), and rats were administered LPS to mimic IP. The mRNA expression of TRAF1 and Sp1 transcription factor (SP1) was analyzed using quantitative real-time polymerase chain reaction. The protein expression of TRAF1, ubiquitin-specific peptidase 7 (USP7), and SP1 was detected by western blotting. Cell viability and apoptosis were assessed using cell counting kit-8 assay and flow cytometry/TUNEL assays, respectively. Interleukin-6 and tumor necrosis factor-α levels were measured by enzyme-linked immunosorbent assays. Reactive oxygen species and malondialdehyde levels were analyzed using fluorescence microscopy and colorimetric assays. The interactions among USP7, TRAF1, and SP1 were identified using co-immunoprecipitation assay, immunofluorescence assay, and dual-luciferase reporter assay. TRAF1 silencing-induced effects were validated in a rat model. Lung tissue pathology was assessed using haematoxylin and eosin assay and Massion assay.

**Results:**

LPS treatment induced apoptosis, inflammation, and oxidative stress of WI-38 cells, however, TRAF1 silencing ameliorated these effects. USP7 stabilized TRAF1 protein expression through its deubiquitinating activity, while TRAF1 overexpression reversed the effects of USP7 silencing in LPS-treated WI-38 cells. In addition, SP1 transcriptionally activated TRAF1 in WI-38 cells. Further, TRAF1 silencing improved lung injury in LPS-induced mice.

**Conclusion:**

Activation of TRAF1 by USP7/SP1 exacerbated the severity of IP, suggesting that targeting TRAF1 may have significant clinical implications for the treatment of IP.

**Supplementary Information:**

The online version contains supplementary material available at 10.1186/s41065-025-00410-x.

## Introduction

Pneumonia is one of the more common respiratory diseases in pediatrics, particularly with a higher incidence rate among the infant and toddler population [1]. Bacteria, viruses, and mycoplasmas are the primary pathogens that cause pneumonia in infants and toddlers, usually spreading through the lungs and causing inflammation [2]. In recent years, due to the change in environmental factors, the incidence of infantile pneumonia (IP) has shown a trend of rising annually [3]. Currently, the treatment for IP mostly involves antiviral and antibacterial therapy. However, with the increasing use of antibiotics, the rising antibiotic resistance of pathogens makes the disease difficult to cure [4]. Thus, conducting comprehensive research into the pathogenesis of IP and identifying more effective therapeutic targets is of paramount importance in improving this condition.

Tumor necrosis factor receptor-associated factor 1 (TRAF1) was initially discovered during the study of tumor necrosis factor receptor 2 signaling complex. It participates in the signaling processes of various tumor necrosis factor receptors, such as tumor necrosis factor receptor-2, latent membrane protein 1 (LMP1), 4-1BB, and cluster of differentiation 40 receptors [5]. There is extensive evidence for its different roles in the progression of lung diseases. For example, TRAF1 promoted tumor invasion of lung cancer by modulating the v-Raf murine sarcoma viral oncogene homolog B (BRAF)/mitogen-activated protein kinase kinase (MEK)/extracellular signal-regulated kinase (ERK) signaling [6]. TRAF1 was expressed in resident lung cells and was responsible for allergic lung inflammation [7]. However, its role in the progression of pneumonia, particularly in IP, as well as the underlying mechanisms, warrant further investigation.

The ubiquitin-proteasome system (UPS) is involved in the regulation of protein silence, protein function, and localization, thereby affecting immune responses, cell cycle progression, and the onset of diseases [8]. Deubiquitinating enzymes (DUBs), as members of the UPS, counteract the function of E3 ligases by cleaving ubiquitin from ubiquitin precursors. Ubiquitin-specific protease 7 (USP7) is a DUB that regulates the stability of numerous target proteins through its deubiquitinating activity [8, 9]. It has been shown to play a significant role in the regulation of protein stability in critical cellular processes including epigenetic regulation and DNA replication [10]. In particular, it has been reported that USP7-dependent mitogen-activated protein kinase 14 (MAPK14) aggravates the progression of pneumonia [11].

Sp1 transcription factor (SP1) stands out as one of the most distinctive transcriptional activators and could bind to the promoter regions of target genes [12]. This binding capacity allows SP1 to modulate the transcription of downstream target genes to influence gene expression profiles critical for various cellular functions. Beyond its role in transcriptional regulation, SP1 is deeply implicated in a multitude of cellular processes, such as cell growth, apoptosis, and angiogenesis [13]. The burgeoning evidence increasingly clarifies that SP1 plays a pivotal role in the progression of diseases, such as atherosclerosis [14], asthma [15] and respiratory tract disorders [16].

Drawing on the comprehensive evidence available, the study posited that TRAF1 was responsible for IP progression, with USP7 and SP1 identified as critical regulators of TRAF1 in this pathological process. To test this hypothesis, the study employed both a rat model and cellular models, with the objective of identifying potential therapeutic targets for the treatment of this condition.

## Materials and methods

### Clinical samples

This study involved 49 children with pneumonia admitted to Northwest Women’s and Children’s Hospital between January and October 2024 as the subjects for analysis. Additionally, 41 healthy children undergoing physical examinations during the same period were included as the control group (healthy control group). Peripheral blood samples were collected from both groups via venipuncture and centrifuged (1,000 g, 10 min, at 4 °C). The supernatant was then stored in an ultra-low temperature refrigerator. The study was approved by the Ethics Committee of Northwest Women’s and Children’s Hospital. Parents or legal guardians of all participants signed a written informed consent form prior to the study. Inclusion criteria for the study comprised (1) children aged between 3 and 10 years diagnosed with pneumonia, presenting with clinical symptoms such as fever, cough, expectoration, and dyspnea; (2) participants were also required to exhibit signs of lower respiratory tract and systemic infection, along with either the presence or absence of rales accompanied by inspiratory stridor; (3) the white blood cell count in their peripheral blood was at or above 4.0 × 10^9^/L; (4) post-imaging examination revealed infiltrative shadows in the lungs of the children. Exclusion criteria included: (1) children with immunodeficiency; (2) children with symptoms of tuberculosis infection or those with bronchial asthma; (3) parents or legal guardians who refused to sign the informed consent form despite being fully informed of the study’s purpose, significance, and detailed execution terms.

### Cell culture and lipopolysaccharide (LPS) stimulation

Human embryonic lung fibroblasts (WI-38, EK-Bioscience) were cultured in Dulbecco’s modified Eagle’s medium (DMEM, EK-Bioscience) added with 10% fetal bovine serum (FBS) and 1% penicillin/streptomycin at 37˚C with 5% CO_2_. WI-38 cells were induced with a pre-configured concentration of LPS (5, 10 and 15 µg/mL, MedChemExpress, Princeton, NJ, USA) at 37 °C for 12 h to mimic an in vitro IP model.

### Cell transfection

Small interfering RNAs (siRNAs) of TRAF1 (si-TRAF1, si-TRAF1#1, and si-TRAF1#2), USP7 (si-USP7, si-USP7#1, and si-USP7#2) and SP1 (si-SP1, si-SP1#1, and si-SP1#2), USP7 overexpression plasmid (OE-USP7), TRAF1 overexpression plasmid (OE-TRAF1), and the matched controls (si-NC and OE-control) were provided by GenePharma (Shanghai, China). WI-38 cells were cultured in DMEM medium containing 10% FBS, maintaining the cell density at 80% or lower confluence. The day before transfection, cell viability needed to be ensured to be stable. Prior to transfection, a microscope was used to observe cell density and morphology. The excess medium was aspirated and replaced with serum-free Opti-MEM (Solarbio, Beijing, China), after which the cells were incubated in an incubator until the transfection mixture was ready to be applied. The plasmids and siRNAs were respectively mixed thoroughly with Lipofectamine 2000 (Thermo Fisher, Waltham, MA, USA) and incubated for 5 min before being added to the cell culture, thus transfecting the WI-38 cells. Cell culture was performed at a temperature of 37 °C for cultivation.

### Cell viability analysis

WI-38 cells were detached from the culture plates, and the cell count within the counting chamber was determined under a microscope. The required volume of solutions was calculated based on a cell density of 5,000 cells per well and added into 96-well plates. After 24 h of culture, the cell status was observed, the supernatant was discarded, and the cells were subjected to transfection and LPS treatment. At 48 h post-treatment, a pre-configured cell counting kit-8 (CCK-8) solution (Beyotime, Shanghai, China) was added, and the plates were incubated in the dark for 2 h. The optical density (OD) value was measured using a microplate reader.

### Cell apoptosis analysis

Cell culture medium was aspirated into centrifuge tubes, and the cells were gently resuspended in phosphate buffer solution before counting. Subsequently, the cells were centrifuged at 1000 g for 5 min to collect them. The cells were then resuspended in Binding Buffer (Solarbio, Beijing, China). Annexin V-fluorescein isothiocyanate (Annexin V-FITC, Solarbio) was added, and the mixtures were gently mixed and incubated in the dark for 15 min. Propidium iodide staining solution (Solarbio) was then added and gently mixed, and the tubes were placed in an ice bath in the dark for 5 min. Flow cytometry analysis was performed within 30 min.

Cell apoptosis was also analyzed using the TUNEL Apoptosis Detection Kit (Yeasen, Shanghai, China). 2 × 10^7^ WI-38 cells were added to poly-L-lysine-coated glass slides and fixed using paraformaldehyde, followed by digestion using Proteinase K solution. The Equilibration Buffer was added to the glass slides, and the samples were incubated with TdT buffer, propidium iodide solution, and 4’,6-Diamidino-2-Phenylindole (DAPI) solution in sequence. Finally, the glass slides were placed under a fluorescence microscope to observe the staining results.

### Enzyme-linked immunosorbent assays (ELISAs)

Commercial kits including Rat myeloperoxidase (MPO) ELISA Kit (SEKR-0073, Solarbio, Beijing, China), Human Interleukin 6 (IL-6) ELISA Kit (AE62763HU, Abebio, Wuhan, China), Rat Interleukin 6 ELISA Kit (AE38205RA, Abebio), Human Tumor Necrosis Factor α (TNF-α) ELISA Kit (AE13959HU, Abebio), and Rat Tumor necrosis factor α ELISA Kit (AE13958RA, Abebio) were used to detect the levels of MPO, IL-6 and TNF-α in the cell supernatant of WI-38 cells, lung tissues or bronchoalveolar lavage fluid according the respective guidebooks.

### Reactive oxygen species (ROS) detection

ROS levels were analyzed using Cellular ROS Assay kit (ab113851, Abcam) according to the guidebook. ROS levels were determined by flow cytometer (Thermo Fisher, Waltham, MA, USA) and fluorescent microscope (Olympus, Tokyo, Japan).

### Malondialdehyde (MDA) analysis

Human MDA ELISA Kit (EH4174, FineTest, Wuhan, China) was used to detect MDA levels. Cell supernatants were harvested and added to detection wells, followed by incubation with biotin-labeled antibody and 3,3’,5,5’-Tetramethylbenzidine. These samples were analyzed using a microplate reader after adding stopping solution.

### Quantitative real-time polymerase chain reaction (qRT-PCR)

Cell supernatants were discarded, and TsingZol (Tsingke, Shanghai, China) was added to culture wells. The mixtures were left to stand for 5 min to ensure complete lysis. Chloroform was then added, and the solutions were allowed to sit for 10 min. The supernatants were collected after centrifugation, to which isopropanol was added. The mixtures were inverted gently to mix and left for another 10 min. The supernatants were discarded once again after centrifugation, and 100% ethanol was added. The precipitates were dissolved in sterile DEPC water and stored at -20°C for later use. According to the instructions of the cDNA Reverse Transcription reagents (Thermo Fisher), the RNA was reverse transcribed into cDNA. Primer sequences were retrieved and downloaded from the national center of biotechnology information genebank database. The obtained cDNA, primers, and SYBR^®^ Premix (TaKaRa, Dalian, China) were mixed according to the manufacturer’s protocol. The mixtures were placed into a fluorescence quantitative PCR instrument (Thermo Fisher), and the experimental data were collected and analyzed through the 2^−∆∆Ct^ method with the normalization to β-actin. TRAF1 5’-GCGCCGAGATGGAGTCATCA-3’ and 5’-GCGATAAAAATCCCCTGGATGGTG-3’, SP1 5’-GTCCGCCCTCTGACCAAG-3’ and 5’-AAGGCACCACCACCATTACC-3’, β-actin 5’-CTTCGCGGGCGACGAT-3’ and 5’-CCACATAGGAATCCTTCTGACC-3’.

### Western blotting assay

After mincing the lung tissue from each group of rats, an appropriate amount of radioimmunoprecipitation assay (RIPA) buffer (Beyotime) was added based on the tissue weight to extract total protein. The RIPA lysis buffer was then transferred to the detection cell wells, and the supernatant was collected. After measuring the protein concentration in each group, samples of equal concentration were prepared. These samples were denatured, and equal volumes of the prepared samples were loaded into the gel wells for electrophoresis. The gels were transferred to polyvinylidene fluoride membranes (GenScript, Nanjing, China) in a transfer apparatus. The membranes were then immersed in blocking solutions for 15 min, followed by washing with phosphate-buffered tween solution. Subsequently, the membranes were incubated with the primary antibodies against TRAF1 (ab300075, 1:1000, Abcam), USP7 (ab108931, 1:5000, Abcam), SP1 (ab124804, 1:5000, Abcam), and β-actin (ab8226, 1:1000, Abcam) as well as the corresponding secondary antibodies (Abcam). The eyoECL Plus (Beyotime) was added for exposure. Finally, the results were saved and analyzed statistically.

### Protein degradation analysis

Transfection with OE-USP7 and control OE-control was performed in six-well plates. After 48 h, the original medium in each well was aspirated, and complete medium containing 200 µg/mL cycloheximide (CHX, Amyjet, Wuhan, China) was added to each well. Cells from each group were collected at 0, 5, 10, 15, and 20 h, respectively. Protein extraction and concentration determination were carried out on the collected cells. The extracted proteins were denatured and subjected to western blotting analysis to validate the effect of USP7 protein expression on the stability of the TRAF1 protein.

### Ubiquitination assays

To directly detect the effect of USP7 on the polyubiquitination of TRAF1 in cell extracts, WI-38 cells were co-transfected with si-USP7 or si-NC. After 48 h, total proteins were collected. All sample concentrations were adjusted and TRAF1 antibody (MA5-15043, 1:50, Thermo Fisher) was incubated with the supernatant. Protein A/G Magnetic Beads (Wanleibio, Shenyang, China) were added to capture the antigen-antibody complexes. The beads were collected using a magnetic separator, washed with lysis buffer, and the complexes were suspended in loading buffer for western blotting. The antibodies included ubiquitin (Ub) antibody (58395, 1:1000, CST, Boston, MA, USA), USP7 antibody (ab108931, 1:5000, Abcam), TRAF1 antibody (ab300075, 1:1000, Abcam), and β-actin antibody (ab8226, 1:1000, Abcam).

### Immunofluorescence assay

Twenty-four hours post-transfection, the cells were digested with trypsin and re-seeded into confocal dishes, after which they were placed in the incubator for further cultivation. 4% paraformaldehyde (Santa Cruz, SantaCruz, California, USA) was added to the dishes to fix the cells for 20 min. Subsequently, 0.5% TritonX-100 solution (Abmole, Shanghai, China) was added to the dishes, and the cells were incubated for 15 min. The primary antibody against USP7 (Thermo Fisher) or TRAF1 (Thermo Fisher) was added to the wells, followed by overnight incubation on a shaker at 4 °C. An appropriate amount of the secondary antibody solution (Thermo Fisher) was then added to the wells using a pipette, and the cells were incubated for 1 h. An adequate amount of antifluorescence quenching mounting medium containing DAPI was added, and the cells were incubated for 5 min. The confocal dishes were then mounted on the stage of a live-cell imaging system, and appropriate parameters were adjusted to observe and capture the images.

### Chromatin immunoprecipitation (ChIP) assay

WI-38 cells were cultured in 10 cm dishes, and formaldehyde (Sigma, St. Louis, MO, USA) was added to crosslink the target protein and the corresponding genomic DNA. Glycine Solution (Wanleibio, Shenyang, China) was added, and the cells were collected into EP tubes. The cell pellets were resuspended in SDS Lysis Buffer (Think-Far Technology, Beijing, China) containing phenylmethanesulfonyl fluoride and incubated on ice for 10 min to fully lyse the cells. The samples were kept on ice, and sonication was performed at 25% power for 3 s on and 17 s off, for a total of 25 min. The supernatants were collected by centrifugation, and ChIP Dilution Buffer (Wanleibio) was added to dilute the sonicated samples. Protein A + G Agarose (Wanleibio) was added to the samples, and the mixtures were slowly rotated at 4 ºC for 30 min. The supernatants were collected by centrifugation, and SP1 antibody (PA5-29165, Thermo Fisher) was added. The control group received incubation with IgG (Wanleibio). The next day, Protein A + G Agarose was added and incubated for 4 h. The precipitates were washed, and the samples were subjected to de-crosslinking. Following this, DNA purification and PCR detection were conducted.

### Dual-luciferase reporter assay

The study constructed the wild-type plasmid carrying the sequence of TRAF1 promoter region and the wild-type reporter gene plasmids with mutated binding sites of SP1 in TRAF1. The cells were seeded in 12-well plates and transfected when the cell density reached approximately 70%. The reporter plasmids were co-transfected into WI-38 cells with si-SP1 and si-NC. Simultaneously, pRL-TK (GenePharma, Shanghai, China) was transfected into the cells. After 48 h of culture, the luciferase activity of the reporter genes was detected using the Dual-Lucy Assay Kit (Solarbio) in a luminometer.

### Animal experiments

Twenty healthy male SD rats (aged 4–6 weeks, weighing 180–200 g, Hunan Slyke Jingda Experimental Animal Co., LTD., Changsha, China) were randomly divided into five groups: Sham, LPS, LPS + As-sh-NC, LPS + Ad-sh-TRAF1, with five rats in each group. The adenoviruses stably expressing sh-TRAF1 and sh-NC were provided by GenePharma Co., LTD. Two groups of rats were injected with As-sh-NC and Ad-sh-TRAF1 via the tail vein. The blank control group and the model group were administered with saline. Three days later, except for the blank control group, the other three groups of rats were given an intranasal instillation of LPS solution (5 mg/kg, MedChemExpress) to replicate the rat model of acute pneumonia. Six hours after instillation, the rats were euthanized. Lung tissue and bronchoalveolar lavage fluid (BALF) samples were collected for subsequent histopathological observation, ELISA kit assays, and western blotting analysis. The animal experiment was permitted by the Animal Research Committee of Northwest Women’s and Children’s Hospital. The study was performed in accordance with the guidelines of the National Animal Care and Ethics Institution.

### Haematoxylin and eosin (HE) staining

The lung tissue sections were heated to 64 °C for 10 min, then cleared and dried using xylene and a range of increasingly concentrated ethanol solutions. The lung tissue sections were hydrated and then stained with hematoxylin solution (Solarbio) for 3 min. An appropriate amount of eosin dye (Solarbio) was added for 3 min, and the sections were successively immersed in descending gradient ethanol solutions and xylene for dehydration and clearing. A drop of neutral resin was added to cover the slides before finally observing and photographing under the microscope. The results were recorded and scored according to the extent of white and red blood cell infiltration in the lung tissue, the count of white and red blood cells in the alveolar space, as well as the amount of fibrin, hyaline membrane, and edema exudate [17].

### Masson staining

The assay was performed using a Masson staining kit (Thermo Fisher). Lung tissue sections from rats were dewaxed and treated with chrome, then washed under running tap water. They were stained with hematoxylin for 5 min and then immersed in Masson solution, followed by immersion in a 2% acetic acid solution for 3 min, 1% molybdate water for 3 min, and stained with aniline blue for 5 min. Afterward, they were immersed in a 0.2% acetic acid solution for 3 min, followed by dehydration and clearing in a series of gradient ethanol solutions and xylene. Finally, the sections were mounted with neutral mounting medium before being observed and photographed under a microscope.

### Lung wet weight/dry weight ratio

After the successful establishment of the model, the lung tissues of the rats (left lung tissue) were excised, the surface liquid was dried, and the weight was measured, with the values recorded as wet weight (W). Then, they were placed in a 60 °C oven for 48 h, after which they were weighed again, with the value recorded as dry weight (D). The W/D ratio of the rat lungs was calculated to assess the degree of pulmonary edema.

### Statistical analysis

The analysis and processing of experimental data were performed using GraphPad Prism 8.0 software. The data were expressed as means ± standard deviations. The differences among multiple groups were tested using one-way analysis of variance (One-Way ANOVA), while the comparison between the two groups was performed using two-tailed Student’s *t*-tests. *P*<0.05 indicated a statistically significant difference.

## Results

### LPS treatment induced WI-38 cell apoptosis, inflammation and oxidative stress

**Fig. 1:**
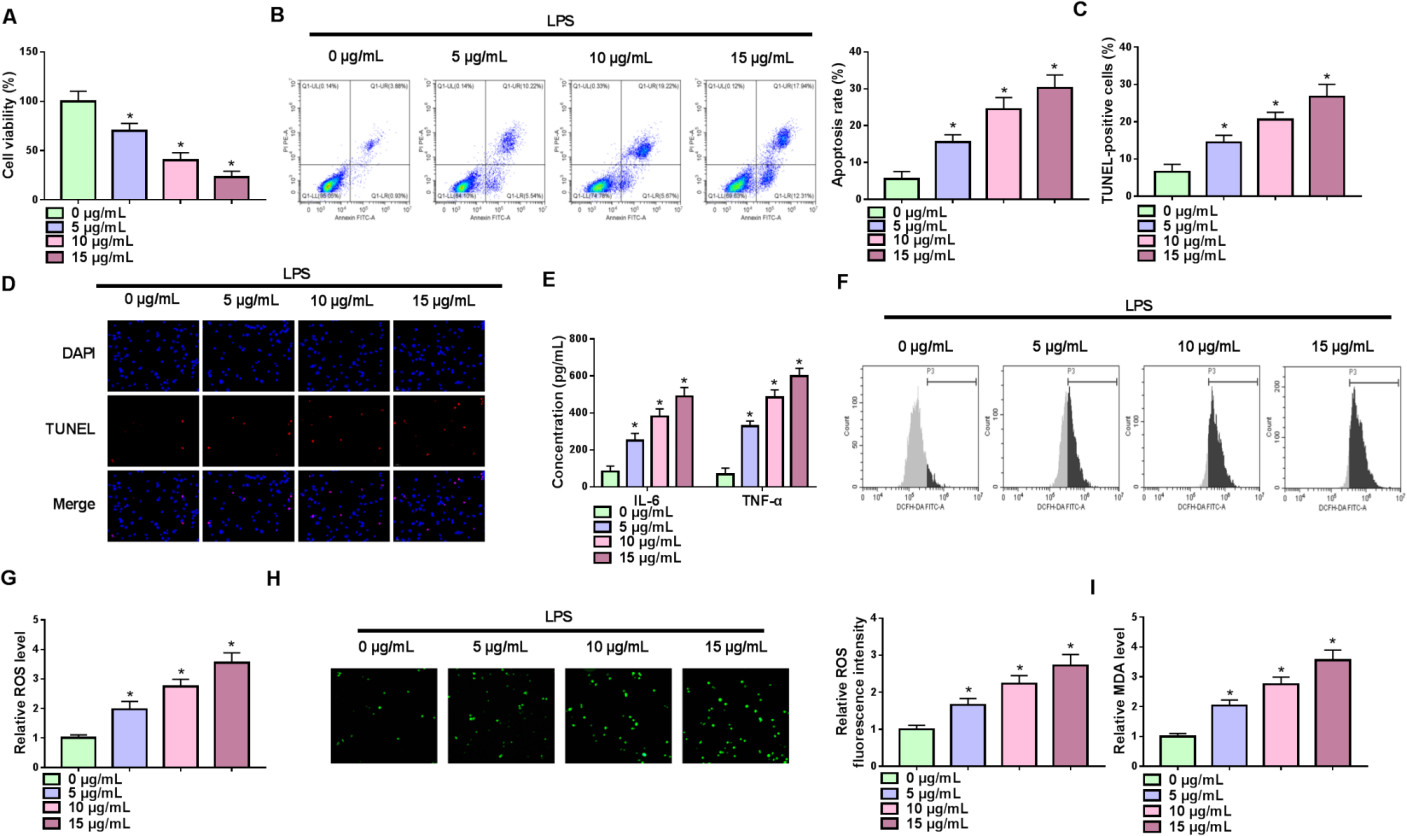
LPS treatment induced WI-38 cell apoptosis, inflammation and oxidative stress. WI-38 cells were treated with various concentrations of LPS (0, 5, 10, and 15 µg/mL). (**A**) Analysis of cell viability. (**B**-**D**) Analysis of cell apoptosis. (**E**) Analysis of IL-6 and TNF-α levels. (**F**-**G**) Analysis of ROS levels. (**H**) Analysis of MDA levels. *P < 0.05

The study treated WI-38 cells using various concentrations of LPS (0, 5, 10, and 15 µg/mL) and then analyzed the subsequent effects of LPS on their biological behaviors. The results showed that LPS treatment inhibited cell viability and induced cell apoptosis in a concentration-dependent manner (Fig. 1A-D). Moreover, the levels of inflammatory factors including IL-6 and TNF-α were increased in a concentration-dependent manner after LPS treatment (Fig. 1E). As shown in Fig. 1F-I, LPS stimulation elevated the levels of ROS and MDA in a concentration-dependent manner. Thus, LPS treatment induced IP-like cell injury.

### TRAF1 silencing ameliorated LPS-induced WI-38 cell apoptosis, inflammation and oxidative stress

**Fig. 2:**
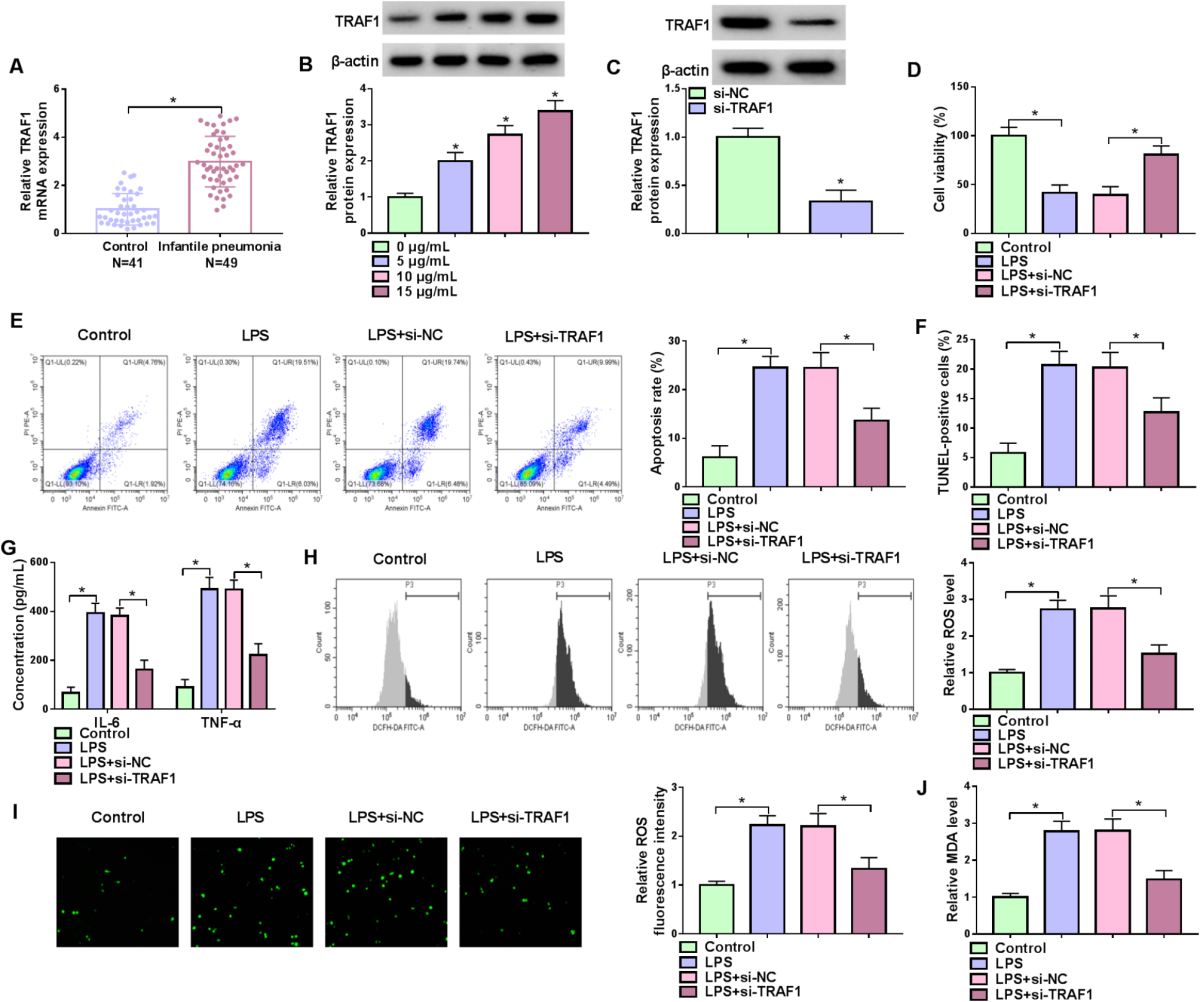
TRAF1 silencing ameliorated LPS-induced WI-38 cell apoptosis, inflammation and oxidative stress. (**A**) Analysis of TRAF1 mRNA expression in the serum of IP patients (*n* = 49) and healthy controls (*N* = 41). (**B**) TRAF1 protein expression was detected in WI-38 cells induced by LPS (0, 5, 10, and 15 µg/mL). (**C**) The efficiency of TRAF1 knockdown was analyzed. (**D**-**J**) WI-38 cells were divided into 4 groups, including Control group, LPS group, LPS + si-NC group, and LPS + si-TRAF1 group. (**D**) Analysis of cell viability. (**E** and **F**) Analysis of cell apoptosis. (**G**) Analysis of IL-6 and TNF-α levels. (**H** and **I**) Analysis of ROS levels. (**J**) Analysis of MDA levels. *P < 0.05

The study then analyzed the role of TRAF1 in LPS-induced WI-38 cell injury. The qRT-PCR analysis revealed its high expression in the serum of IP patients than in healthy controls (Fig. 2A). The western blotting assays also showed its high protein expression in LPS-induced WI-38 cells (Fig. 2B). Subsequently, the study transfected TRAF1 siRNA and its control si-NC into WI-38 cells and LPS treatment was performed. The data presented by western blotting assay showed that TRAF1 protein expression was significantly decreased after the transfection with si-TRAF1, si-TRAF1#1 and si-TRAF1#2, especially transfection with si-TRAF1 (Fig. 2C and Figure S1A). si-TRAF1 was used for the following study. We also discovered that LPS treatment-induced inhibition in cell viability and promotion in cell apoptosis was attenuated after TRAF1 silencing (Fig. 2D-F). Moreover, the transfection with TRAF1 siRNA counteracted the LPS treatment-induced increases in the levels of IL-6, TNF-α, ROS and MDA (Fig. 2G-J). The study also analyzed the effects of TRAF1 overexpression on LPS-induced WI-38 cell injury. As shown in Figure S2A-E, LPS treatment-induced inhibitory effect on cell viability and promoting effects on cell apoptosis, IL-6, TNF-α, and ROS levels were enhanced after TRAF1 overexpression. Thus, TRAF1 knockdown protected against LPS-induced cell apoptosis, inflammation and oxidative stress.

### USP7 stabilized TRAF1 protein expression through its deubiquitinating activity

**Fig. 3:**
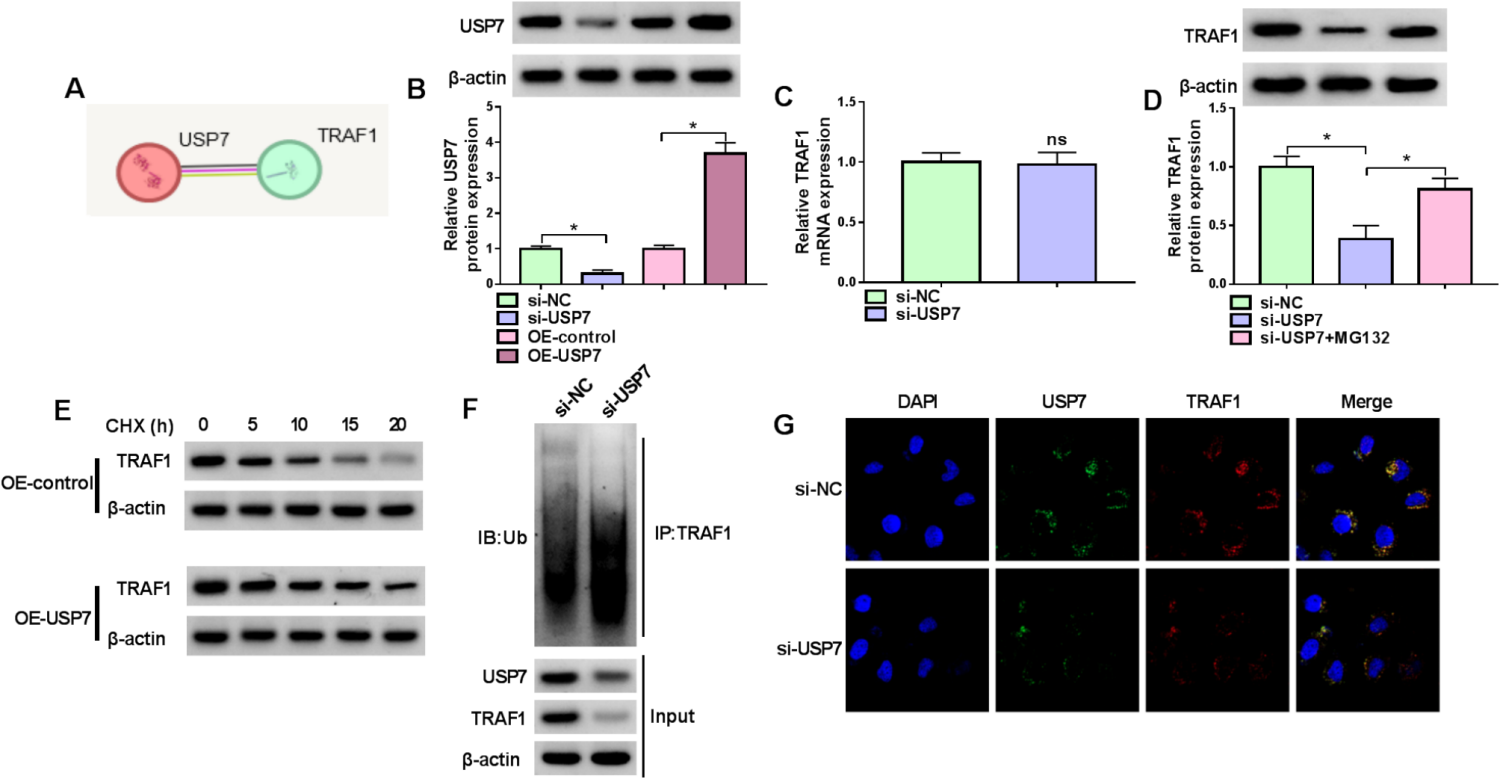
USP7 stabilized TRAF1 protein expression through its deubiquitinating activity. (**A**) The STRING database predicted the association of USP7 with TRAF1. (**B**) The efficiency of USP7 silencing or overexpression was analyzed. (**C**) The effect of USP7 silencing on TRAF1 mRNA expression. (**D**) WI-38 cells were transfected with si-USP7 or si-NC and treated with MG132, and TRAF1 protein expression was analyzed. (**E**-**G**) The association of USP7 and TRAF1 in WI-38 cells. ns: not significant. *P < 0.05

The STRING database predicted that USP7 potentially interacted with TRAF1 (Fig. 3A), indicating the possible regulatory role of USP7 in TRAF1. The study then synthesized USP7 siRNAs (si-USP7, si-USP7#1 and si-USP7#2) and USP7 overexpression plasmid to analyze the association of USP7 and TRAF1. Their efficiency in downregulating or upregulating USP7 protein expression is shown in Fig. 3B and Figure S1B. si-USP7 was selected for subsequent study due to its highest inhibitory effect on SP1 expression. Subsequently, we discovered that USP7 silencing did not affect the mRNA expression but significantly inhibited its protein expression (Fig. 3C and D). However, the USP7 siRNA-induced inhibitory effect on TRAF1 protein expression was relieved after treatment with MG132 (Fig. 3D). The CHX assay showed that the stabilization of TRAF1 protein was enhanced after USP7 overexpression (Fig. 3E). Moreover, we discovered that USP7 silencing increased the levels of ubiquitinated TRAF1 (Fig. 3F). Further, the results showed that USP7 was colocalized with TRAF1 in the cytoplasm of WI-38 cells, and the phenomenon was weakened after USP7 silencing (Fig. 3G). Thus, USP7 induced the deubiquitination of TRAF1 in WI-38 cells.

### TRAF1 overexpression attenuated USP7 silencing-induced effects in WI-38 cells subjected to LPS treatment

**Fig. 4:**
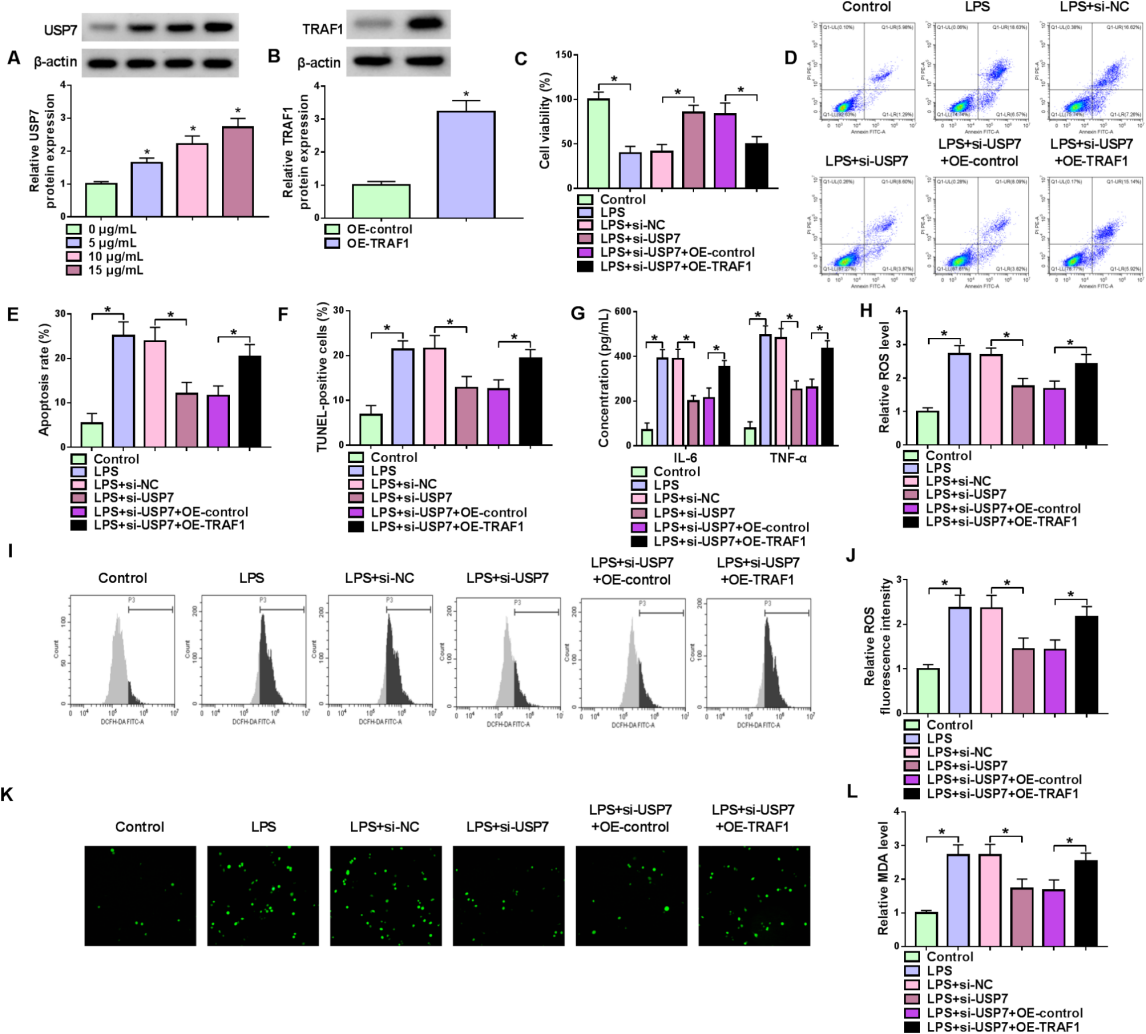
TRAF1 overexpression attenuated USP7 silencing-induced effects in WI-38 cells subjected to LPS treatment. (**A**) The effect of LPS on USP7 protein expression. (**B**) The efficiency of TRAF1 overexpression was determined. (**C**-**L**) WI-38 cells were divided into 6 groups, including Control group, LPS group, LPS + si-NC group, LPS + si-USP7 group, LPS + si-USP7 + OE-control group, and LPS + si-USP7 + OE-TRAF1 group. (**C**) Analysis of cell viability. (**D**-**F**) Analysis of cell apoptosis. (**G**) Analysis of IL-6 and TNF-α levels. (**H**-**K**) Analysis of ROS levels. (**L**) Analysis of MDA levels. *P < 0.05

The study subsequently explored the association of TRAF1 and USP7 in regulating LPS-induced WI-38 cell injury. The result revealed that LPS treatment increased USP7 protein expression in a concentration-dependent manner (Fig. 4A). Then, the study transfected USP7 siRNA, TRAF1 overexpression plasmid, and the respective controls into WI-38 cells, followed by LPS treatment. The efficiency of TRAF1 overexpression is shown in Fig. 4B. We discovered that USP7 silencing promoted cell viability and inhibited cell apoptosis in the cells, but these effects were relieved after TRAF1 overexpression (Fig. 4C-F). The results also showed TRAF1 overexpression attenuated the decreases in the levels of IL-6, TNF-α, ROS and MDA induced by USP7 silencing (Fig. 4G-L). Thus, USP7 silencing protected against LPS-induced cell apoptosis, inflammation and oxidative stress by regulating TRAF1 expression.

### SP1 transcriptionally activated TRAF1 in WI-38 cells

**Fig. 5:**
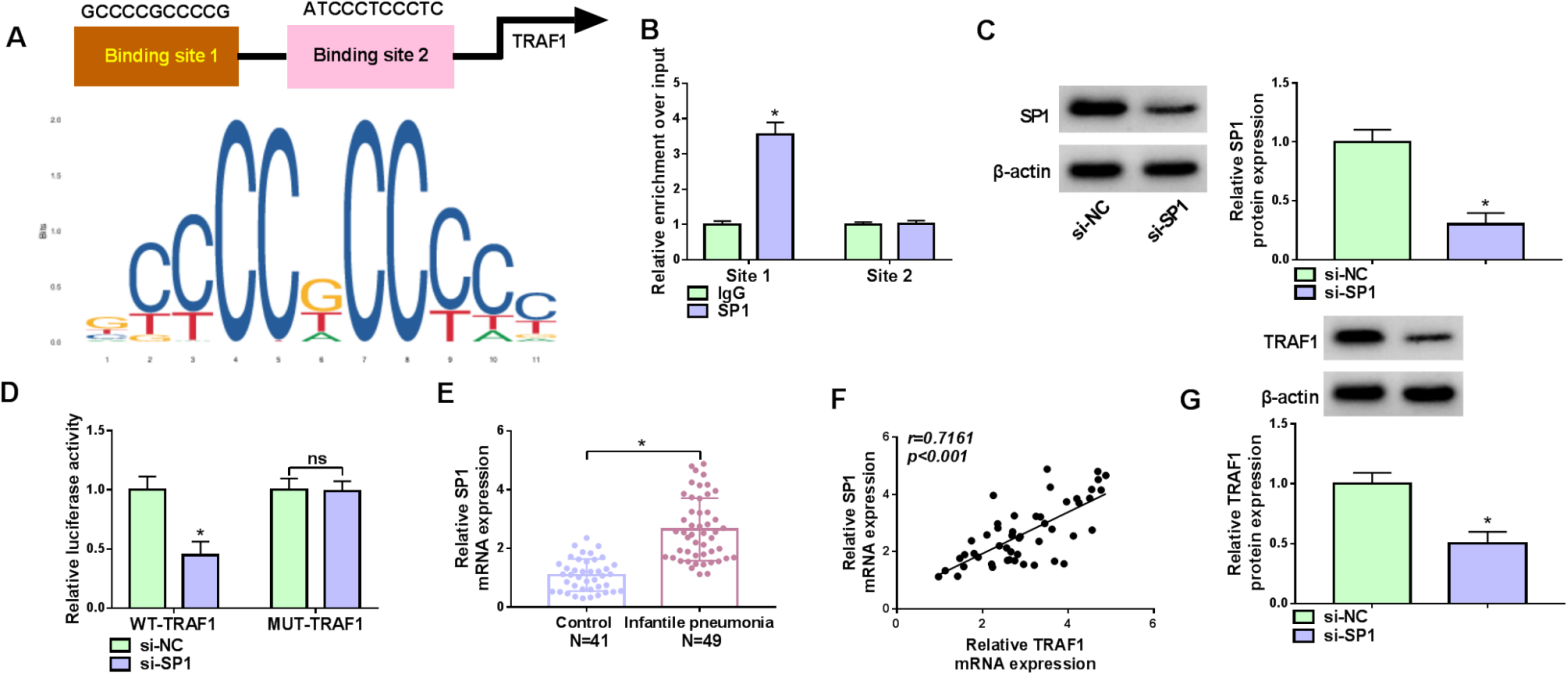
SP1 transcriptionally activated TRAF1 in WI-38 cells. (**A**) The schematic illustration showed the binding sites of SP1 in the promoter region of the TRAF1 mRNA. (**B**) ChIP analysis of the association of SP1 with TRAF1. (**C**) The efficiency of SP1 knockdown was analyzed. (**D**) Luciferase reporter analysis of the association of SP1 with TRAF1. (**E**) SP1 mRNA expression in the serum of IP patients and healthy controls. (**F**) The correlation of SP1 with TRAF1 in the serum of IP patients. (**G**) The effect of SP1 knockdown on TRAF1 protein expression. *P < 0.05, ns: not significant

Through the prediction of the jaspar database, we discovered that the promoter region of the TRAF1 mRNA contained two binding sequences (site 1 and site 2) of SP1 (Fig. 5A). The ChIP assay showed that site 1-containing DNA fragment was significantly enriched by the SP1 antibody (Fig. 5B). Subsequently, the study transfected TRAF1 reporter plasmids into WI-38 cells with SP1 siRNAs (si-SP1, si-SP1#1, and si-SP1#2) or si-NC and detected the luciferase activity of the cells. As shown in Fig. 5C and Figure S1C, the efficiency of SP1 siRNAs in reducing SP1 expression was analyzed by western blotting assay. si-SP1 was chosen for the following study due to its highest inhibitory effect. The co-transfection of si-SP1 with WT-TRAF1 significantly inhibited the luciferase activity, however, the co-transfection of SP1 siRNA with MUT-TRAF1 did not affect the luciferase activity (Fig. 5D). In addition, the result showed that SP1 expression was upregulated and was positively correlated with TRAF1 expression in the serum of IP patients (Fig. 5E and F). Moreover, TRAF1 protein expression was inhibited after SP1 knockdown (Fig. 5G). Thus, SP1 induced the transcriptional activation of TRAF1 in WI-38 cells.

### TRAF1 silencing ameliorated the lung injury of LPS-induced rats

**Fig. 6:**
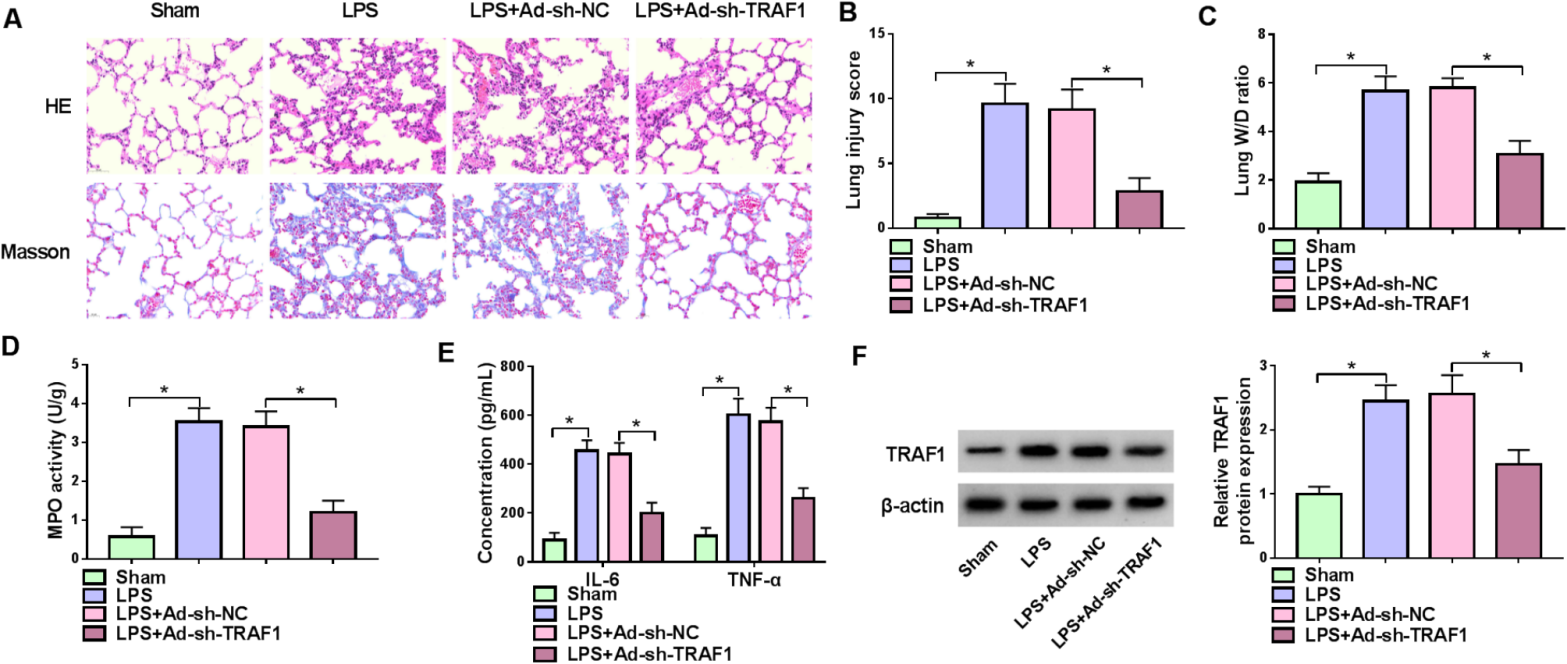
TRAF1 silencing ameliorated the lung injury of LPS-induced rats. Rats were administrated with adenovirus stably expressing TRAF1 siRNA or si-NC and then treated with LPS. After 6 days, the rats were euthanatized, and lung tissues and bronchoalveolar lavage fluid were harvested for the following analysis. (**A** and **B**) Analysis of the pathological feature of lung tissues. (**C**) Analysis of W/D ratio. (**D**) Analysis of MPO levels in lung tissues. (**E**) IL-6 and TNF-α levels in bronchoalveolar lavage fluid. (**F**) Analysis of TRAF1 protein expression in lung tissues. *P < 0.05

The protective effect of TRAF1 silencing against WI-38 cell injury was further identified through a rat model. To achieve this, the study administrated rats with adenovirus stably expressing TRAF1 shRNA or sh-NC and then treated with LPS. Through the HE staining analysis (Fig. 6A), we discovered that the lung tissue in the control group exhibited normal cell structure, with intact alveoli and bronchi, and no infiltration of inflammatory cells. By comparison, the model group displayed significantly thickened alveolar walls, loss of alveolar structure, and a substantial infiltration of inflammatory cells. In contrast to the model group, the Ad-sh-TRAF1 treatment group showed some degree of improvement, with less thickening of the alveolar walls, a more preserved alveolar structure, and a reduced level of inflammatory infiltration. In addition, the Massion staining result showed that in comparison with the control group, the LPS group exhibited thickened alveolar walls, and there was a visible accumulation of collagen around them. This condition was ameliorated after treatment with Ad-sh-TRAF1 (Fig. 6A). The analysis for lung injury score is shown in Fig. 6B. We also observed that LPS treatment increased the ratio of lung W/D, promoted MPO activity and increased the levels of IL-6, TNF-α and TRAF1, whereas these effects were relieved after TRAF1 silencing (Fig. 6C-F). Thus, TRAF1 silencing protected against LPS-induced lung injury of rate.

## Discussion

The primary methods for treating IP currently include antiviral, antibacterial, as well as symptomatic supportive treatments [18]. However, due to the prolonged and excessive use of antibiotics, pathogenic bacteria have gradually become resistant, which not only makes the treatment of the disease more difficult but also increases the risk of treatment failure [19]. Therefore, further understanding the underlying mechanisms of IP is essential for the treatment of IP. LPS is a major endotoxin component in the cell walls of Gram-negative bacteria, and it plays a critical role in inflammatory responses. Recent studies have found that LPS can significantly stimulate various types of cells, including monocytes, macrophages, and endothelial cells, to produce a large amount of chemokines and inflammatory factors. The high expression of these chemokines and inflammatory factors leads to the intensification of local inflammatory responses, which in turn triggers a series of pathological changes [20, 21]. Therefore, LPS is often used as an important tool in the study of various inflammatory diseases, including IP, to induce cell inflammation models. The study analyzed the role of TRAF1 in IP using LPS-induced WI-38 cells and LPS-stimulated rats as well as the underlying mechanism. The study showed that TRAF1 silencing ameliorated LPS-induced WI-38 injury and lung injury of rats, and the mechanism involved the regulation of USP7 and SP1 in its expression.

Previous evidence has shown that TRAF1 aggravates LPS-induced human bronchial epithelial cell apoptosis and inflammation through interaction with circ_0038467 and miR-545-3p in neonatal pneumonia [22]. We analyzed its role in IP using LPS-induced WI-38 cells and LPS-induced rats. Herein, we discovered that TRAF1 expression was upregulated in the serum of IP patients and LPS-induced WI-38 cells. Similar to the above-mentioned papers, our study reported its promoting effects on IP progression. We discovered that its knockdown attenuated LPS-induced promoting effects on WI-38 cell apoptosis, inflammation and oxidative stress. Moreover, TRAF1 silencing also ameliorated LPS-induced lung injury in rats. As reported, LPS treatment increased TRAF1 expression, and TRAF1 silencing protected Ana-1 murine macrophages against LPS-induced inflammation by inactivating the AKT and nuclear factor kappa B (NF-Κb) signaling [23]. Another paper revealed that TRAF1 activated c-Jun N-terminal kinase (JNK) pathway to aggravate LPS-induced lung injury [24]. Collectively, the above data indicated that TRAF1 exacerbated the progression of IP by regulating the AKT and NF-κB signaling as well as JNK pathway.

Our research demonstrated that USP7, known for its role in protein stabilization, enhanced the expression of TRAF1. As previously documented, USP7 interacted with MAPK14, thereby playing a role in the regulatory mechanisms of esculin in the context of LPS-induced pneumonia [11]. Our data extended these findings by showing that the knockdown of USP7 improved LPS-induced apoptosis, inflammation, and oxidative stress through the regulation of TRAF1 in WI-38 cells. Furthermore, our study revealed that SP1, a transcription factor, stabilized TRAF1 mRNA expression. This is particularly relevant in the context of mycoplasma pneumoniae infection, which has been shown to induce SP1 phosphorylation, leading to a decrease in RECK expression and subsequent promotion of matrix metalloproteinase-9 (MMP-9) secretion [25]. Our data also showed an upregulation of SP1 expression in the serum of IP patients, suggesting a potential role for SP1 in the disease’s progression. Moreover, we found that SP1 bound to the promoter region of TRAF1, thereby inducing its mRNA expression. In comparing our findings with previous studies, our work provides a more detailed molecular mechanism linking TRAF1 activation to the exacerbation of IP, specifically through the USP7/SP1 axis. This adds a layer of complexity to our understanding of the pathophysiology of the disease and opens up new avenues for therapeutic intervention.

The present work has several limitations that should be acknowledged. Firstly, the study was conducted in vitro using cell lines and in vivo using a rat model, which may not fully recapitulate the complex pathophysiology of IP in humans. In the case of IP, the immune system of infants is very different from that of rats or cell lines in terms of development, immune response, and susceptibility to pathogens. The differences in lung structure, immune system maturity, and the way infants interact with their environment compared to adult rats could limit the translation of findings to a clinical setting. This means that while the mechanisms observed may be biologically relevant, their actual contribution to the disease in humans might be different. Thus, the findings may require further validation in clinical studies involving human subjects. Additionally, the study focused on the role of TRAF1, USP7, and SP1, but the interaction with other molecular pathways and the broader immune response to LPS could also influence disease severity and may not have been fully explored. Without exploring these interactions, the true contribution of TRAF1 to pneumonia severity might be overestimated or underestimated.

The study provides evidence that the activation of TRAF1 by the USP7/SP1 axis contributes to the exacerbation of IP severity. The findings suggest that modulating the activity of TRAF1, USP7, and SP1 could provide a novel approach for managing this common and potentially severe respiratory infection in infants. Specifically, the application of antisense oligonucleotides or inhibitors directed against USP7 (such as P5091 and HBX 19818) and SP1 (like mithramycin A) could facilitate the degradation of TRAF1, thereby reducing the severity of IP.

## Electronic supplementary material

Below is the link to the electronic supplementary material.

Supplementary Material 1

Supplementary Material 2

## Data Availability

The datasets used and/or analyzed during the current study are available from the corresponding author upon reasonable request.
