# Supplementary figures and images for: Activation of TRAF1 induced by USP7/SP1 exacerbates the severity of infantile pneumonia
Source: Hereditas. 2025 Mar 22;162:44. doi: 10.1186/s41065-025-00410-x (PMC11929315; doi:10.1186/s41065-025-00410-x)

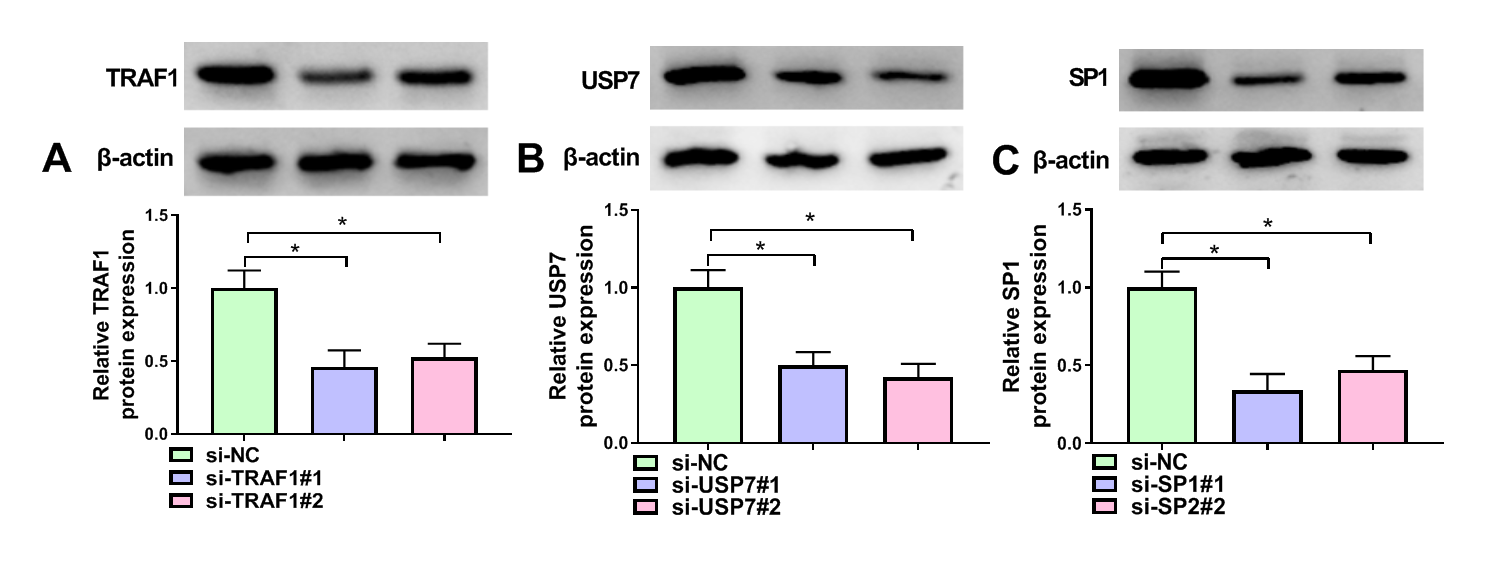

Supplement: Supplementary file 1 — Supplementary Material 1 [file 41065_2025_410_MOESM1_ESM.tif]

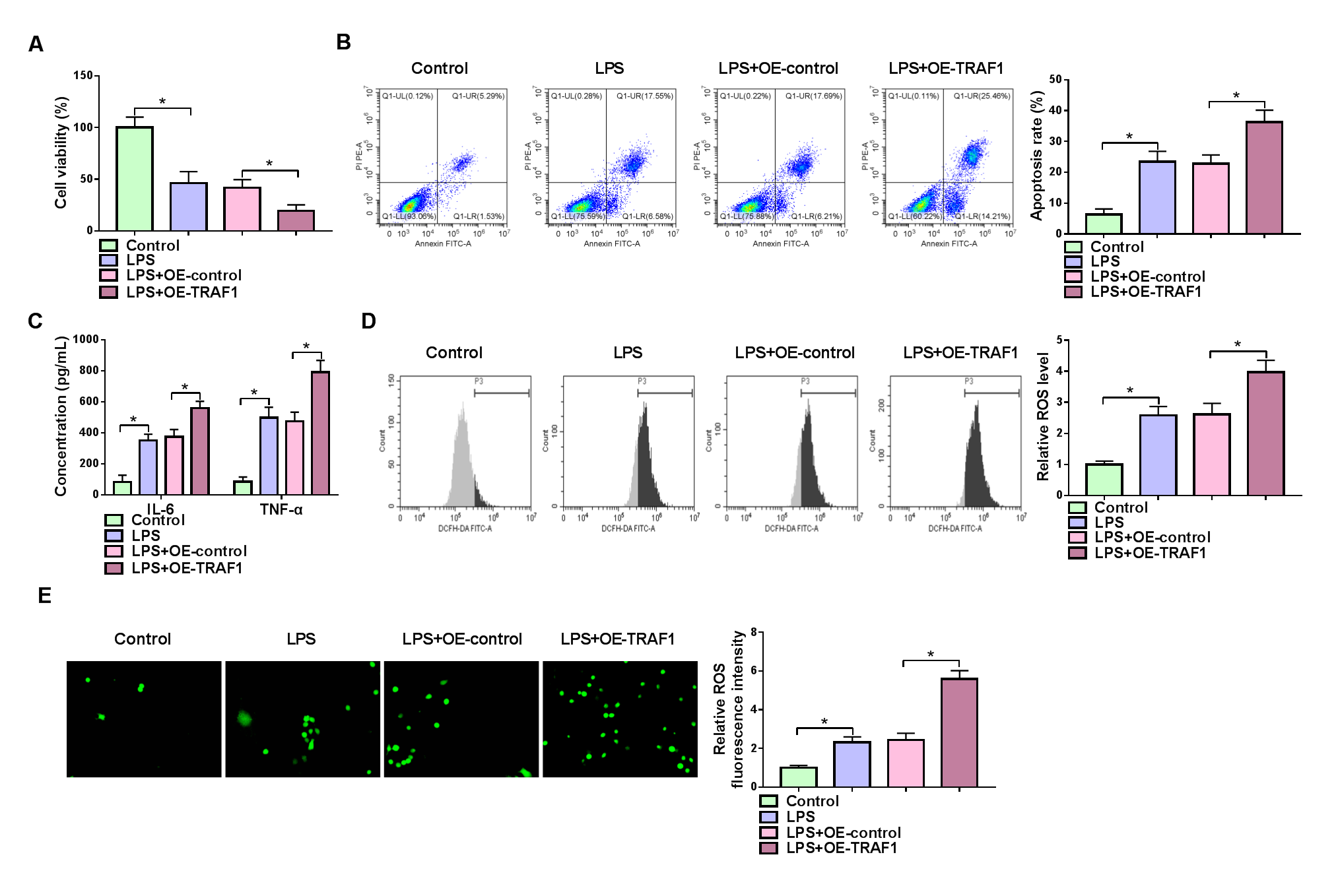

Supplement: Supplementary file 2 — Supplementary Material 2 [file 41065_2025_410_MOESM2_ESM.tif]
